# Chronic Dislocation of the Thumb Carpometacarpal Joint: A Case Report

**DOI:** 10.7759/cureus.38168

**Published:** 2023-04-26

**Authors:** Grayson C Kelmer, Andrea H Johnson, Justin J Turcotte, Alexander Shushan

**Affiliations:** 1 Medical School, Campbell University School of Osteopathic Medicine, Lillington, USA; 2 Orthopedic Research, Luminis Health Anne Arundel Medical Center, Annapolis, USA; 3 Orthopedic Surgery, Luminis Health Anne Arundel Medical Center, Annapolis, USA

**Keywords:** suture bridge, ligament repair with augmentation, open reduction, dorsoradial ligament, chronic dislocation, thumb carpometacarpal joint

## Abstract

Dislocation of the thumb carpometacarpal (CMC) joint is a rare injury; chronic CMC dislocation can lead to significant disability. Traditionally, surgical intervention has focused on the reconstruction of the anterior oblique ligament, though more recently there has been more focus on the dorsoradial ligament. Consideration of both ligaments during CMC joint reconstruction is important to optimize functional outcomes.

A 59-year-old male presented with a subacute/chronically dislocated CMC joint of the thumb. Open reduction with pin fixation and dorsoradial ligament repair and augmentation was chosen to restore the stability of the joint. Joint reduction without subluxation was successfully maintained. By 12 weeks postoperatively, there were no remaining major restrictions to activity.

Repair of the dorsoradial ligament with augmentation and pinning is a viable approach for surgical management of subacute/chronic dislocation of the thumb CMC joint.

## Introduction

Dislocation of the thumb carpometacarpal (CMC) joint is a rare occurrence, composing less than 1% of hand injuries [1]. Traumatic dislocation is usually caused by the application of axial force to a flexed thumb, resulting in dorsal displacement and potentially ligamentous injury [2]. Many separate ligaments are known to form the thumb CMC joint capsule and help stabilize the articulation [3]. However, the exact number of distinct ligaments is debated, ranging from three to 16 and complicated by differences in nomenclature [3-5].

It is theorized that the anterior oblique (beak) ligament and the dorsoradial ligament (DRL) are the two most important ligaments for joint stability [3,4]. Historically, cases of thumb CMC joint instability have favored reconstruction of the anterior oblique ligament (AOL) [4,6]. More recent literature supports the DRL as being equally if not more essential in stability, both stiffer and thicker than the AOL [4,6-8]. Consideration of both of these ligaments during CMC joint reconstruction is important in optimizing the surgical approach and functional outcomes. In this case, we present a subacute/chronic thumb CMC dislocation successfully repaired via open reduction and repair of the DRL with suture tape augmentation and pinning.

Consent for the publication of this case study was obtained from the patient.

## Case presentation

Clinical findings

A 59-year-old male was presented to the clinic with right-hand pain after an incident approximately one month prior to the visit where he slipped and fell off a ladder onto his hand. At the time, the patient experienced swelling and pain. The patient used over-the-counter medications to manage his injury but had continued pain and presented for evaluation. Physical examination showed deformity and tenderness at the base of the right thumb. Sensation and capillary refill were intact. Significant past medical history included diabetes mellitus and hyperlipidemia.

Imaging findings

**Figure 1:**
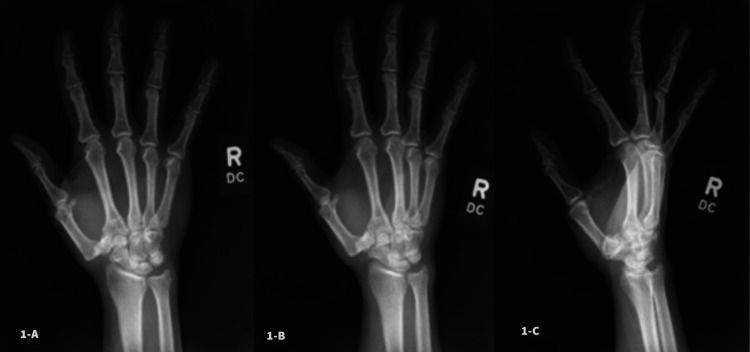
Preoperative X-rays in posteroanterior (Fig. 1-A), oblique (Fig. 1-B), and lateral (Fig. 1-C) views demonstrating dislocation of the thumb CMC joint

An X-ray and MRI obtained the week prior to the presentation were reviewed. The X-ray revealed a dislocation of the thumb CMC joint (Figure 1).

The MRI showed no evidence of osseous fragments and confirmed dislocation of the metacarpal base relative to the trapezium. Mild adjacent interstitial edema and subcortical edema along the radial thumb metacarpal base were present. Subtle edema was also present in the distal flexor pollicis. Both forms of imaging supported a diagnosis of subacute/chronic CMC dislocation of the right thumb.

Assessment and surgical plan

Clinical and radiographic findings were reviewed with the patient, and surgical intervention was recommended. Risks, benefits, and alternatives were discussed. The patient agreed to open reduction and pin fixation of the CMC joint with possible DRL reconstruction using palmaris autograft versus native ligament repair with augmentation using the internal brace technique. Suggested alternatives included a fusion of the CMC joint and ligament reconstruction and tendon interposition (LRTI). Consideration was also given to AOL reconstruction using flexor carpi radialis (FCR). DRL reconstruction with pinning was chosen after a discussion regarding the surgical options. In the case of failure, conversion to LRTI or fusion could still be considered.

Surgical procedure

**Figure 2:**
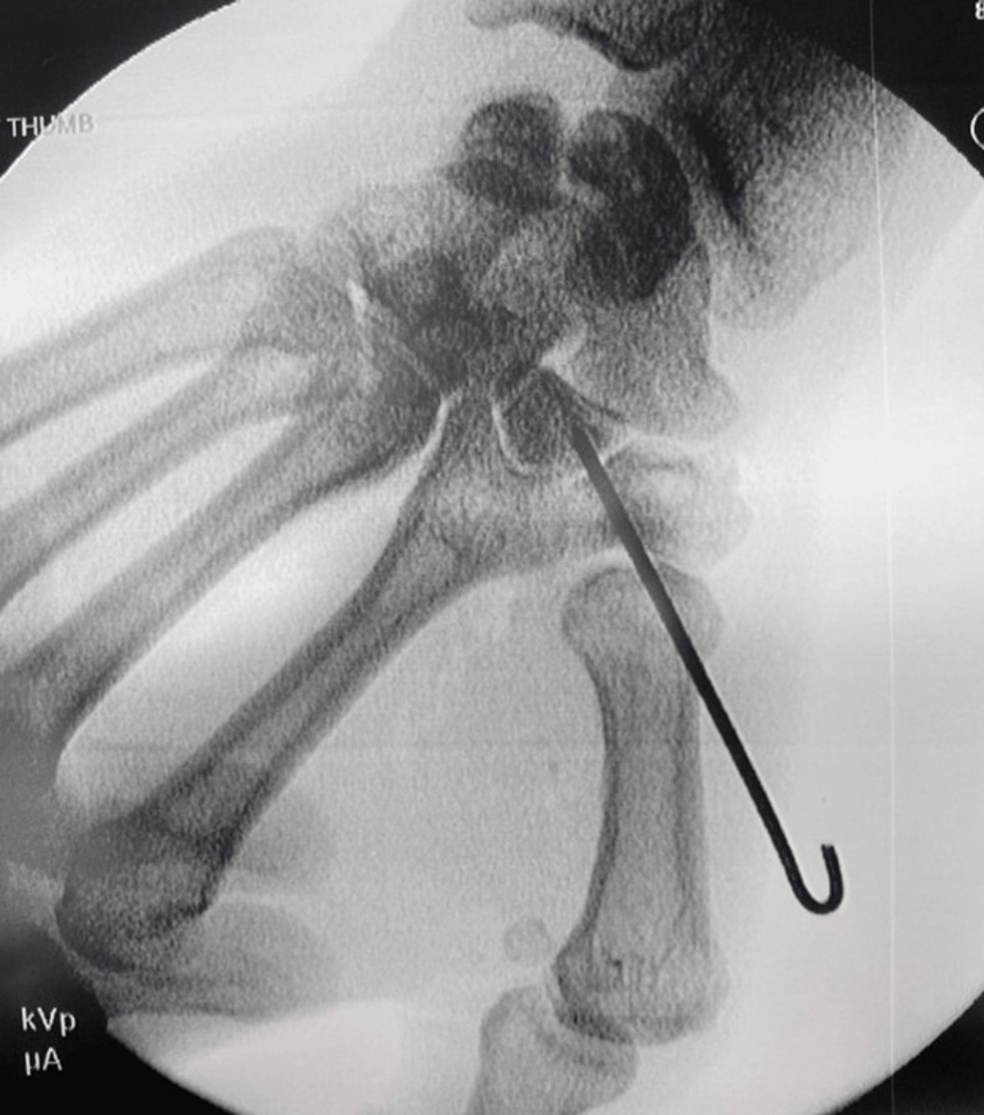
Post-reduction intraoperative film

The patient returned 16 days after the initial consultation for surgical intervention. A dorsal incision was made, and blunt dissection was carried down to the dorsal metacarpal and first dorsal compartment. Dissection proceeded off the metacarpal base and into the joint to achieve better mobility of the metacarpal and visualize the dislocated joint. A freer elevator was placed into the joint and used as a lever to open up the joint and dissect the capsule off the dorsal aspect of the trapezium and the joint was anatomically reduced. After verification of full reduction via X-ray, a K-wire was introduced and driven across the trapezium (Figure 2).

An X-ray was used to refine the reduction before the pin was fully driven into the trapezium. Excellent stability was achieved. Multiple X-ray projections were again used to confirm near anatomic joint alignment.

**Figure 3:**
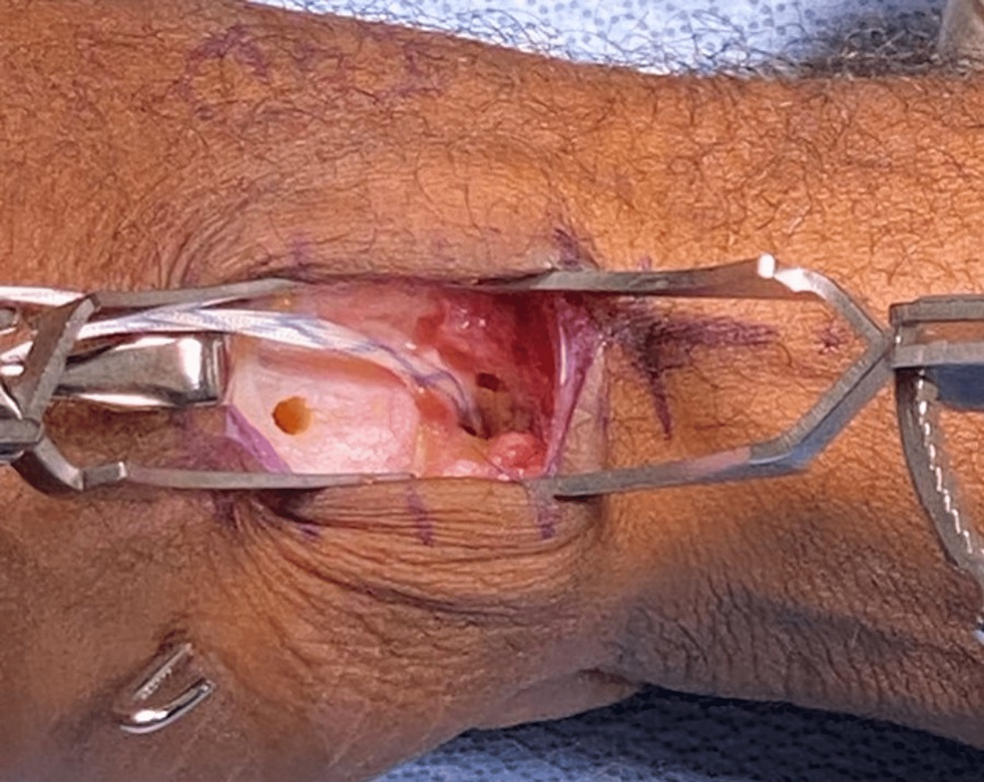
Suture anchor with attached suture tape placed in the trapezium

Following open reduction, and noting the quality of the native DRL, DRL repair with internal brace augmentation was selected. Two K-wires were placed, one into the trapezium and one into the metacarpal base, at the level of the DRL. The metacarpal K-wire was placed in the same location as would be required for an LRTI. X-rays were taken to confirm positioning. Bone tunnels were drilled, and an anchor with suture tape was placed into the trapezium (Figure 3).

**Figure 4:**
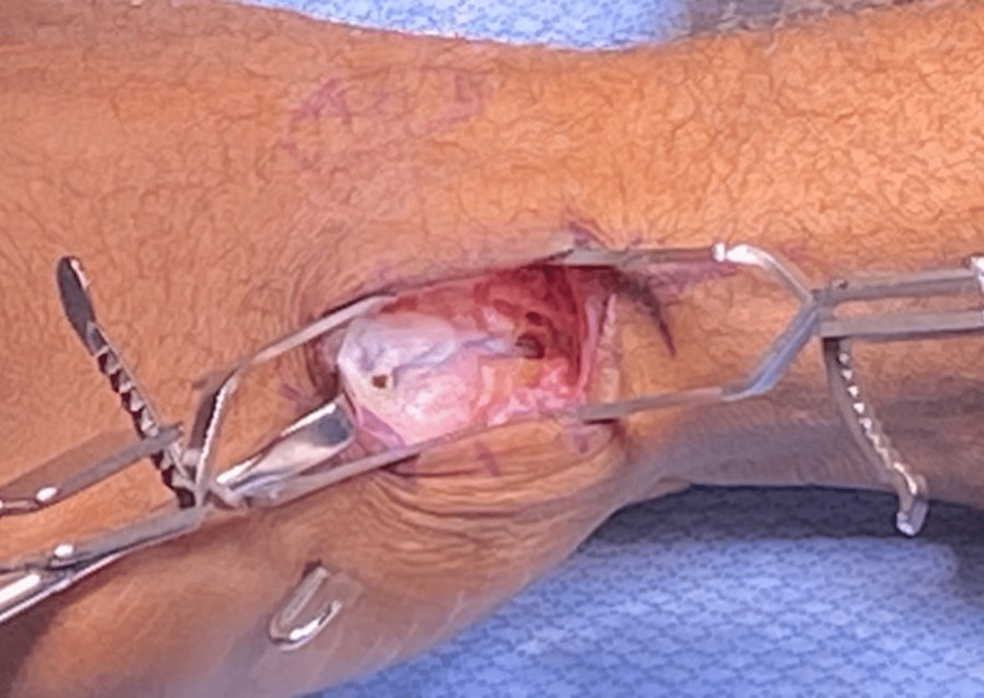
Final suture bridge construct in place

Suture tape was placed and doubled over onto the metacarpal base, and a second anchor was placed (Figure 4).

Final X-rays were taken to confirm proper anchor placement and reduction. The joint capsule was repaired with the native DRL, the wound was closed, and a thumb spica splint was applied.

Postoperative follow-up and outcomes

**Figure 5:**
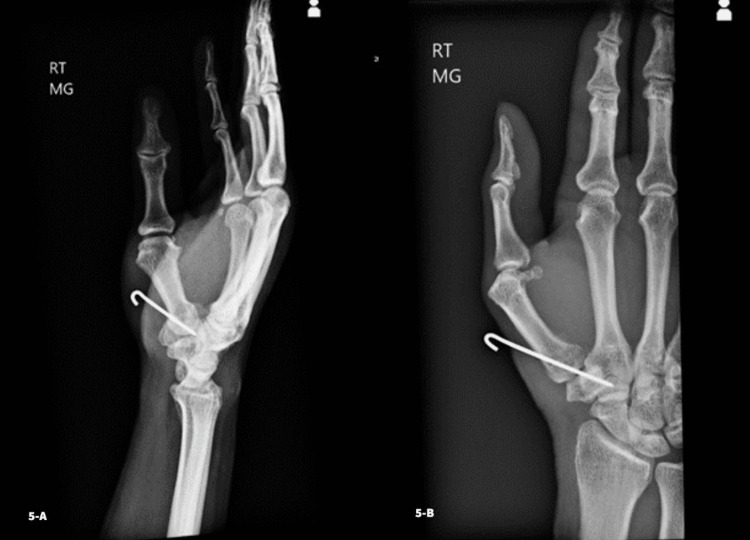
Lateral (Fig. 5-A) and posteroanterior (Fig. 5-B) views two weeks postoperatively following open reduction and pin fixation

Two weeks postoperatively, X-rays were taken and reviewed for joint reduction and pin position (Figure 5).

**Figure 6:**
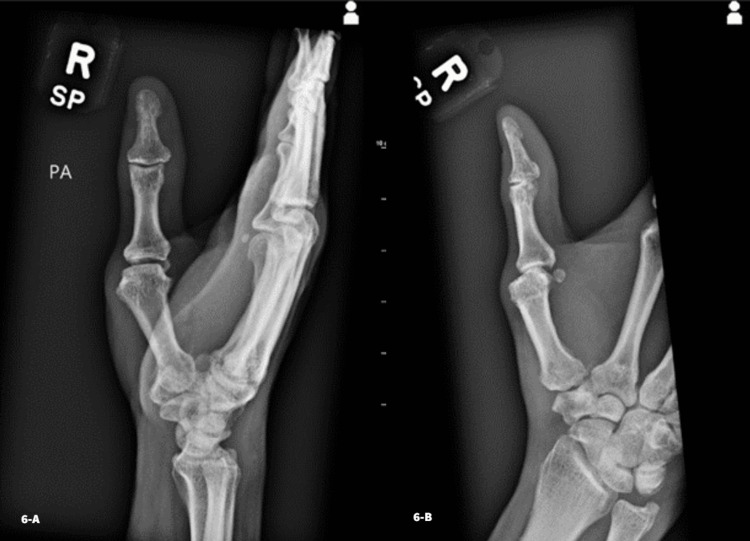
Lateral (Fig. 6-A) and posteroanterior (Fig. 6-B) views 12 weeks postoperatively showing maintained reduction without evidence of subluxation

The patient was transitioned to a thumb spica cast. At four weeks postoperatively, another set of X-rays were taken. Pin removal was performed, and a new spica cast was applied with freedom of the interphalangeal joint. Six weeks postoperatively, imaging was repeated, confirming stability of the joint reduction, and the patient was placed in a custom thermoplastic thumb spica splint. At 12 weeks postoperatively, X-rays demonstrated maintenance of joint reduction without subluxation (Figure 6).

Upon examination at the 12 week visit, the patient noted stiffness of the joint but was able to oppose his thumb to the distal interphalangeal joint of the fifth digit. Continuation of physical therapy was recommended to improve motion in the thumb and wrist. Splinting was discontinued, and no further restrictions were placed on the patient.

## Discussion

Optimal treatment options for acute and chronic dislocations of the thumb CMC joint are still being explored [2,9-11]. Acute dislocations can be managed with closed reduction and casting, but nonsurgical intervention is not always sufficient [2,10,11]. Most cases, whether acute or chronic, are managed with ligament reconstruction, and many involve removal of the trapezium [10]. Surgical intervention is usually required in cases of longer-term instability [9]. Open reduction, pinning, K-wire fixation, tendon repair or reconstruction, and fusion are techniques that have been used to resolve chronic dislocations, all of which were either performed or considered in the management of this case.

While CMC dislocation of the thumb is a rare injury, there is far more documentation of acute cases than chronic [10]. Contact sports, falls, and motorcycle accidents are common causes of dislocation, and patients generally present to the emergency department or clinic with pain and swelling shortly after such incidents [10,12]. However, dislocation of the thumb CMC joint can be missed. In significant accidents with multiple injuries, a full evaluation of the thumb CMC joint may not be prioritized [2]. X-rays in anteroposterior, oblique, and lateral views, as well as stress radiographs, can be necessary to properly diagnose the dislocation [2,12]. Thus, three situations that may result in progression to a chronic CMC dislocation of the thumb are a delayed initial visit of the injured patient, as seen in our case; a failure to diagnose the dislocation upon first presentation of a hand injury [9,13]; or prioritization of evaluation of more severe injuries following a traumatic event [2].

A missed diagnosis of CMC dislocation leading to chronic injury has been described by McCarley and Foreman [13]. A 41-year-old male suffered a hand injury and was seen in the emergency department the following day. Limited X-rays did not clearly demonstrate any abnormalities. Two years later, the patient presented for further evaluation and was diagnosed with dorsal dislocation of the thumb CMC joint [13]. The prolonged nature of this injury resulted in significant soft tissue contracture and scar tissue, eliminating both closed and open reduction as options for surgical management. Resection of the first metacarpal base and distal trapezium with fusion was necessary [13]. This case further supports a full thumb series and a stress view as the standard for radiography in suspected cases of thumb CMC dislocation [2,12,13]. It also emphasizes the importance of early management of thumb CMC dislocations, as prolonged dysfunction of the joint can limit options for surgical intervention.

Other cases describing chronic dislocations of the thumb CMC joint classify chronic as anywhere from 30 days to over a year [14,15]. Mazhar et al. reviewed nine cases of thumb CMC dislocation, three of which were chronic. These three cases had dislocation durations of 30 days, 60 days, and eight months [15]. All patients were reconstructed using FCR via the Eaton-Littler technique. In a comparison of postoperative factors, the patients with chronic dislocation had better functional outcomes than some of the patients with acute dislocation [15]. Overall, multiple surgical techniques are viable for the repair of chronic thumb CMC dislocations of various duration. Dislocations past one year may benefit from additional consideration of dysfunctional changes in CMC soft tissues for optimization of the functional recovery period.

## Conclusions

As knowledge of the thumb basal joint grows, and the importance of the DRL is better appreciated versus the AOL, treatment for subacute/chronic dislocations can be rethought. Rather than LRTI or AOL repair/reconstruction, DRL repair or reconstruction could play a role in the treatment of these difficult cases. In the present case, we apply the biomechanical knowledge of the importance of the DRL to consider novel repair/reconstruction options for a subacute/chronic CMC dislocation. This case supports open reduction and pin fixation with repair and augmentation of the DRL as a viable approach for surgical management of subacute/chronic thumb CMC joint dislocation. Further reports and studies are necessary to fully explore optimal treatment for chronic CMC joint dislocations of various durations.
